# Lekking as collective behaviour

**DOI:** 10.1098/rstb.2022.0066

**Published:** 2023-04-10

**Authors:** Akanksha Rathore, Kavita Isvaran, Vishwesha Guttal

**Affiliations:** ^1^ Centre for Ecological Sciences, Indian Institute of Science, Bengaluru, Karnataka, Bangalore 560 012, India; ^2^ Ecology of Animal Societies, Max Planck Institute of Animal Behavior, 78467 Konstanz, Germany

**Keywords:** self-organization, emergent patterns, mate-choice, sexual selection, evolutionary dynamics, territoriality, clustering, collective motion

## Abstract

Lekking is a spectacular mating system in which males maintain tightly organized clustering of territories during the mating season, and females visit these leks for mating. Various hypotheses—ranging from predation dilution to mate choice and mating benefit—offer potential explanations for the evolution of this peculiar mating system. However, many of these classic hypotheses rarely consider the spatial dynamics that produce and maintain the lek. In this article, we propose to view lekking through the perspective of collective behaviour, in which simple local interactions between organisms, as well as habitat, likely produce and maintain lekking. Further, we argue that interactions within the leks change over time, typically over a breeding season, to produce many broad-level as well as specific collective patterns. To test these ideas at both proximate and ultimate levels, we argue that the concepts and tools from the literature on collective animal behaviour, such as agent-based models and high-resolution video tracking that enables capturing fine-scale spatio-temporal interactions, could be useful. To demonstrate the promise of these ideas, we develop a spatially explicit agent-based model and show how simple rules such as spatial fidelity, local social interactions and repulsion among males can potentially explain the formation of lek and synchronous departures of males for foraging from the lek. On the empirical side, we discuss the promise of applying the collective behaviour approach to blackbuck (*Antilope cervicapra*) leks—using high-resolution recordings via a camera fitted to unmanned aerial vehicles and subsequent tracking of animal movements. Broadly, we suggest that a lens of collective behaviour may provide novel insights into understanding both the proximate and ultimate factors that shape leks.

This article is part of a discussion meeting issue ‘Collective behaviour through time’.

## 1. Introduction

Organisms exhibit a wide range of mating strategies not only across species but also within a species or even at the population level [1–3]. Typically, mating strategies involve more than just a pairwise interaction between a male and a female. A mating interaction between a particular male and a particular female is the outcome of many social interactions, e.g. males competing aggressively to monopolize a female, males displaying to attract the attention of a female, females competing for a high-ranking male, and females copying the choice of other females. In other words, the chance that a male and a female mate depends not only on the traits of the focal pair but also, crucially, on interactions with other males and females in the population. An extreme form of such a mating system is a lek—where both males and females show an exceptionally wide range of social interactions leading to mating.

**Figure 1.**
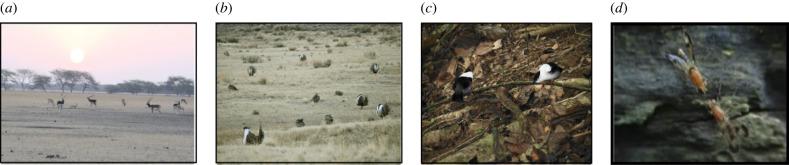
Examples of lek-mating systems. (*a*) Blackbuck (*Antilope cervicapra*) lek at Velavadar National Park (picture credit: Shruti Hegde). (*b*) Greater sage-grouse (*Centrocercus urophasianus*) lek by USFWS Pacific Southwest Region (CC BY 2.0 by Jeannie Stafford USFS). (*c*) White-bearded manakin (*Manacus manacus*) males displaying on a twig (CC BY-NC 2.0 by jpc.raleigh). (*d*) Druid-fly (*Clusia tigrina*) engaged in mating (CC BY-SA 3.0 by Pristurus, 2011).

Lekking is a rare but extensively studied phenomenon to understand sexual selection and mating strategies [4–7]. In this mating system, territorial males aggregate on traditional breeding grounds and defend territories that are devoid of any resources (figure 1). Males perform elaborate mating displays and directly compete with one another for access to mates. Females visit the lek and move between territories to sample males or in response to the behaviour of males [8]. When females visit a territory, males display to the approaching female while also showing aggression to chase away other males. While the mating interactions on the lek may happen over a range of a few seconds to hours, the formation and maintenance of the lek is a much longer process. In addition, the spatial scales of interactions range from within a single territory to an entire lek, involving a large number of individuals. Therefore, a lek is a site of multiple social interactions, in which interactions change as a function of spatial and temporal scales and eventually contribute to the mating success of individuals.

In this article, we argue that lekking may be viewed as a collective behaviour system driven by a myriad of social interactions that change over time. We take inspiration from the field of collective animal motion; numerous studies have shown that investigating simple local interactions among organisms can provide novel insights into the large-scale patterns and functions of animal societies [9–12]. We assert that a similar approach, which considers lekking as a collective phenomenon and therefore studies lekking systems at fine spatial and temporal resolutions, could offer a unique perspective to our understanding of the proximate and ultimate dynamics of lekking. We hypothesize that local interactions among individuals amplify to produce many patterns at the global level, e.g. lek formation, mate choice and skewed mating success. We illustrate the broad principles of this hypothesis using a computational model, and high-resolution spatio-temporal data from blackbuck, a charismatic antelope species showing a spectacular form of lekking behaviour.

### (a) Hypotheses of lek evolution

We begin by summarizing some basic ideas about lek evolution for the uninitiated reader. Lekking is puzzling as it appears to be associated with high costs for males, via high competition for mating with no energetic resources [13–18]. Some of the classic hypotheses of lek evolution focus on the role of female movement. When female home ranges are large, males may find it difficult to track them [1,2]. Hence, males occupy territories at the intersection of female home ranges to, potentially, increase their chances of encountering females. Since many males try to occupy these overlapping home range locations, an aggregation of territories might arise in these areas—leading to the formation of leks. This is called the ‘hot-spot’ hypothesis [19–21]. Alternatively, the clustering of territories could be driven by female movement before mating. If we assume that females move randomly between territories before mating, males may increase their chances of encountering females by clustering; clustered territories are more likely to retain the female, sort of acting like a ‘black-hole’ [22–24]. The name 'black-hole', as a curious reader would have guessed, is inspired by the black holes of astrophysics!

According to a broad class of explanations under the ‘female-choice hypothesis’, females prefer to mate with males that are a part of aggregations rather than solitary males. Several reasons have been proposed for such a preference, including that clusters reduce predation risk during the mating process, females face less harassment by males when present on the lek, the probability of finding a high-quality mate increases with male clustering or that clusters provide a low-cost mate-sampling opportunity for females [4,25–27]. Another hypothesis is called the ‘hotshot hypothesis’; here, if females have a preference for particular males, other males may try to form territories around these preferred males called ‘hotshots’ and try to court visiting females [28–30].

Explicit spatial thinking reveals that some of these hypotheses are not always mutually exclusive. For example, in the black-hole model, fine-scale female random movement (scanning nearby territories) drives the clustering of territories; in contrast, in the hot-spot model, large-scale home range movement patterns of females may drive male clustering. In other words, the spatial scale of female movement is the main distinguishing feature of black-hole versus hot-spot models of lek evolution. Further, in the hot-shot model where females prefer specific males, certain courtships spill over to neighbouring ‘satellite’ territories. Thus, the fine-scale movement strategies of satellite males—e.g. to intercept and modify the movement of females visiting the ‘hotshot’ male—on the lek are important to investigate. In addition, there are large-scale movements between leks by females and satellite males that can impact the stability and mating consequences on the lek. There is also empirical evidence for this line of argument, pointing to the possibility of different mechanisms acting at different spatial scales even within the same population/species to ultimately give rise to lek dynamics [22,25,31–35].

### (b) Collective behaviour: a brief summary

Animal collectives exhibit fascinating patterns at the group level that may have functions such as consensus and shared decisions, navigation and foraging, improved vigilance and predator escape [9,36–42]. Studies in the field of collective animal behaviour focus on the relationship between individual interactions and the emergence of group-level patterns and functions. Spatially explicit consideration of animal interactions has been a key for both theoretical and empirical studies in the field of collective animal behaviour [10,12,42]. Agent-based models of collective motion consider each agent’s behaviour in terms of properties of spatial location, motion and their interaction with neighbours. Depending on the ecological context, the behaviours of organisms are modified. For example, individual variations (heterogeneity) can shape various sorting within groups, group structures and merge-split dynamics among groups [40,43,44]. Broadly, these studies reveal that even simple interactions between organisms can explain many properties of collective motion [11,44–46]. On the other hand, group-level properties are not merely a sum of individual properties. Thus, they highlight the importance of explicitly accounting for how local interactions shape emergent group-level patterns.

To be able to test predictions of collective behaviour models, empirical approaches collect data on the movement of organisms at high spatial and temporal resolutions. Such data help us construct fine-scale trajectories of each organism [12,45,47]. By using this, one can decipher fine-scale interactions between organisms, as well as test predictions about novel group-level properties such as decision-making and information transfer. For example, analyses of high-resolution trajectories of fish, birds, insects, etc. reveal that organisms do indeed follow relatively simple rules of interactions, broadly as suggested by theory, to produce various macroscopic patterns [42,45–48].

## 2. Lekking as a spatial collective phenomenon

We now elaborate on how the formation and maintenance of a lek can be viewed as a spatial collective phenomenon arising from local interactions between individuals at various spatio-temporal scales.

### (a) Local interactions, lek formation and maintenance

Here, we hypothesize that the proximate causes for lek formation can be explained by local interactions among males.

1. *Spatial fidelity:* Males show a preference to form territories on a traditional mating ground. Thus, in a proximate sense, males are attracted to this site and start marking territories on the mating ground.
  Box 1. **Lek as collective behaviour and spatial ecological system.**
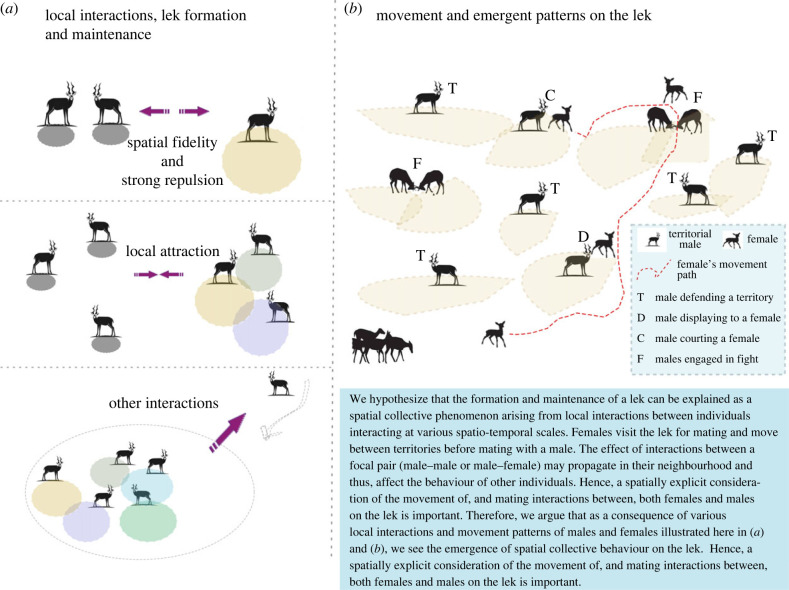
2. *Mid-range attraction:* To explain the tight clustering of territories observed in many species, we hypothesize local attraction interactions between males (box 1).
3. *Small-range repulsion:* While males are forming (clustered) territories, they also show a local repulsion towards each other, typically via marking and defending territories from intruding males.
4. *Other interactions:* While the aforementioned three factors contribute towards lek formation, we hypothesize that several other interactions could be at play for the maintenance of leks. On a lek, two or more neighbouring males may together chase away intruder males, a possible cooperative interaction, thus defending both of their territories on the lek. Such a cooperative interaction could contribute to the benefits of territory clustering and help explain the maintenance of leks. In addition, certain ‘complex’ interactions among lek members may also be necessary for males to continue to hold their territories as they regularly depart and return to the lek.

### (b) Movement and mating interactions on the lek

Females visit the lek for mating and move between territories before mating with a male. There are competing hypotheses on whether females move on the lek to sample mates, assess the quality of males and thus exercise mate choice, or females move largely as a consequence of various interactions on the lek. We argue that for either of these hypotheses, spatially explicit consideration of the movement of, and mating interactions between, both females and males on the lek is important. We propose to classify the movement patterns and mating interactions on the lek as follows:

1. *Male movement within their territories (local movement):* Males move within their territories to court and mount the females present on their territories. They may also move to the boundary of their territory to display to or chase away intruding males.
2. *Male excursions outside their territories (lek-level movement):* Males also move outside their territories in short spurts to attract females and bring them to their territory. Movement spurts may also result in a disruption of courtship on another male’s territory.
3. *Female movement in response to males’ behaviour:* Females may move between various male territories in response to males’ behaviour, such as chases by males, which can eventually result in a mating that is not necessarily driven by mate choice. Female movement within a territory can also result in the disruption of the courtship of a male with another female, e.g. when a female shows aggression towards the courting male or female.
4. *Female movement for mate sampling:* Females may actively follow decision rules such as conspecific cueing, preference for central territories, phenotype preference, etc., resulting in mate sampling or mate choice dynamics.

### (c) Emergent patterns on the lek

We now explain how local interactions on the lek change with time and how they may influence collective patterns on the lek. To do so, we consider the most-typical male–male interaction dynamic on the lek: males show aggressive behaviours towards nearby individuals (local repulsion), which help them maintain their territories. Under a few scenarios described later, we argue that the local repulsion behaviour changes with time to a local copying interaction. We speculate about both the proximate (i.e. how these local interactions may produce larger-scale patterns) and ultimate factors (i.e. functions) that may underlie such behaviours.

1. *Synchrony and coordination in departures:* When a male leaves the lek for feeding, local repulsion among males may change to a local attraction, or more specifically the copying of a neighbour’s movement behaviour. This may lead to a synchronous departure of males for feeding; the number of individuals and/or the spatial extent of this synchronous feeding departure will depend on the strength of copying interactions. We hypothesize that such a change from aggression to a local movement copying (or coordination) in the context of feeding may evolve in response to trade-offs between factors such as: (i) energetic costs of staying on the lek without feeding for long and (ii) potential loss of mating opportunities when one goes out of the lek for feeding.
2. *Competition and cooperation among males:* When an intruder male enters a lek, especially in the central areas, males nearby may switch from their local repulsion behaviour to local coordination of movement among neighbours. This may lead to a higher chance of chasing the intruder away. In other words, local aggression or competition changes over time to a local cooperative behaviour. We speculate that such local behaviour may be crucial to maintain the stability of the entire lek during the breeding season. We hypothesize that this behaviour may be selected for by females’ preference for relatively stable neighbourhoods on the lek for mating [49–51]; a neighbourhood where intruders are not present will have less aggression, thus attracting females for mating.
3. *Spatial dynamics and mating success:* When a female enters a lek, local repulsive interactions between nearby males may change in myriad ways. For instance, two males may increase their aggression and fiercely fight to access the female. At the same time, other nearby males may avoid each other but engage in a courtship display to attract the female. On the other hand, when a male–female pair is engaged in courtship, neighbours may try to disrupt this. Females may explore such a neighbourhood or many such neighbourhoods on the lek and choose a male to mate with based on a variety of their own decision rules (see §3 and table 1). Therefore, while mating is an interaction between a pair, the outcome of mating interactions for both males and females at the population level is a function of local neighbourhood interactions on the lek. An example of such an emergent pattern could be the well-known characteristic of leks where we observe a highly skewed mating success among males, i.e. some of the males receive most of the matings, which can result in strong sexual selection [32,52,53].
  Put together, these examples lend credence to the argument that simple local interactions and associated fine-scale movement of organisms could help us better understand the lek as a collective phenomenon. This naturally raises the question of how one can utilize the analysis of fine-scale movement to test different hypotheses. In table 1, we describe how emergent patterns and their alternative hypotheses can be potentially verified via fine-scale movement patterns. We elaborate on these ideas further in the next two sections.

**Table 1.** We list some plausible emergent patterns, alternative hypotheses that could explain these patterns and corresponding local decision rules that organisms could follow. Finally, in the last column, we provide how movement trajectories are predicted to differ between various hypotheses.

emergent pattern	hypothesis	local decision rule	predicted movement trajectories
Male departure from leks	(I) Independent/random departures	Leave when feeding is required.	Low or no correlation in departure time stamps in a neighbourhood.
	(II) Synchronized departures	Copy your immediate neighbours when they leave for feeding.	Highly correlated departure time stamps in a neighbourhood.
Chasing away intruders by lekking males	(I) Independent chasing	Chase away intruders when they enter your territory area.	Low or no correlation in the trajectories of neighbours chasing away intruders in a neighbourhood.
	(II) Cooperative chasing	Copy your neighbours in chasing behaviour in the local neighbourhood.	Highly correlated trajectories while chasing away intruders.
Mating-success pattern on the lek arising from female mating behaviour	(I) Random mate sampling	Choose a male randomly on the lek.	Random movement pattern with no discernible feature.
	(II) Preference for central territories	Go to central territories and mate.	Movement trajectory leading to central territories and then going outside the lek.
	(III) Mate choice copying	If another female is present, go to the same location and mate.	Movement trajectory and location of the male chosen by a newly arriving female correlates strongly with those of a female already present on the lek.

## 3. An agent-based model

We now illustrate via a simple agent-based model of how local interactions can produce some basic emergent patterns of the lek. Specifically, we focus on the formation of the lek and synchronous feeding departures of lekking males. We then discuss various directions for model extensions and how to link them with data.

### (a) Model and results

At the beginning of the lekking season, males are attracted towards the breeding ground, leading to the formation of the lek. We can model this process with a few basic rules of movement in a continuous two-dimensional landscape, where individuals update their velocities and positions at discrete time steps. The first rule is that individuals move towards the breeding centre at a constant speed, with some error (i.e. noise) in the direction of motion. Without loss of generality, we assume the centre of the breeding ground to be the origin of our spatial coordinates. The second rule is that individuals repel each other when they are within a short distance *R*_*r*_ of each other. Between these two rules, the repulsion rule is of higher priority, i.e. when an individual is trying to move towards the breeding centre but encounters another within a distance *R*_*r*_, only the repulsion rule is implemented.

**Figure 2.**
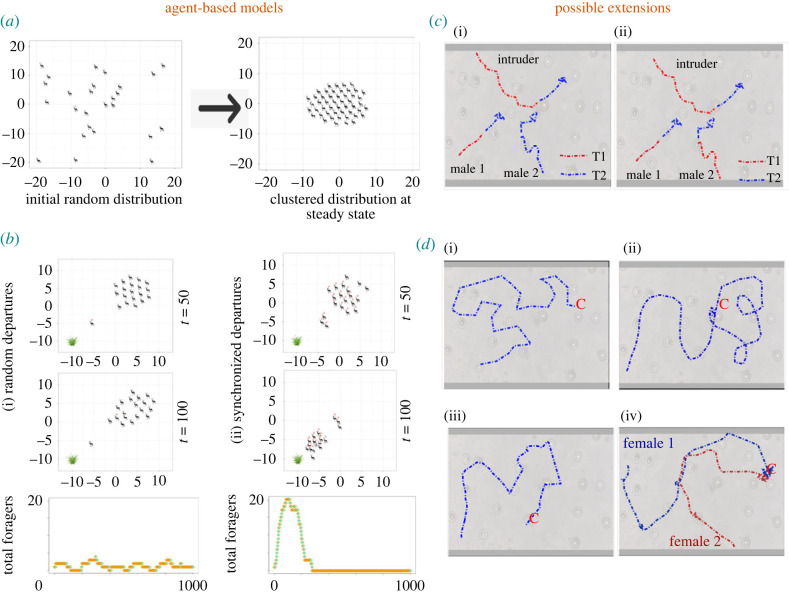
(*a*) Lek formation: output from an agent-based model showing the male-clustering arising from site fidelity and local repulsion interactions. (*b*) Foraging departures: patterns of departures of lekking males for agent-based models having (i) no social interactions vs (ii) copying interactions among neighbours. It can be seen in the time series that the copying model predicts that the number of foragers shows a steep curve on the time axis. (*c*) Territory defence: hypothetical trajectories for two examples of how resident lekking males chase an intruder. Red dotted lines represent trajectories before the start of the chase. Blue trajectories show the trajectory after the male begins chasing the intruder. While (i) shows that two males had asynchronous independent chasing, (ii) represents coordinated chasing. (*d*) (i–iv) Hypothetical trajectories of females corresponding to various female mate-sampling strategies on the lek: (i) random sampling, (ii) best of ‘*N*’, (iii) threshold-based, and (iv) choice copying.

We place individuals randomly in a landscape and simulate the above rules over many discrete time steps. Figure 2*a* illustrates the initial state and the steady state of these simulations, clearly demonstrating that the formation of a lek requires a few basic rules.

Next, we model one of the emergent patterns described in table 1: (coordinated) male departure for foraging. Since the lekking ground contains no foraging resources, males typically depart from the lek to forage. In the case of blackbuck, for example, foraging activity varies through the day with peaks in the morning and evening. During such a foraging peak, lekking males typically leave the lek to feed and return once the foraging episode is completed. All males complete one such foraging episode during a given foraging activity peak. To model this, we first consider the lek thus formed from the above set of rules. In the foraging time window, we assume that each individual on the lek can temporarily switch to a foraging state, thus foregoing its spatial fidelity. We assume that the switch happens stochastically, with a probability *p*_*f*_ at each discrete time step. An individual in the foraging state is attracted towards a nearby foraging site (or a water hole). After reaching the foraging site, the individual switches back to the lekking state and thus returns to the lek arena. For the purpose of this exercise, we do not model within-lek spatial fidelity of individuals when they return.

We now consider two possible ways that foraging departures from the lek may happen. A first possibility is where each individual switches to a foraging state independent of the foraging state of other males on the lek. A second possibility invokes social behaviour; specifically, we assume that, at each discrete time step, each individual in the lekking state may switch to the foraging state at a probability that is proportional to the number of neighbours in the foraging state. Once an individual switches to the foraging state, it moves towards the foraging site and returns to the lekking site as described earlier, without any further social interactions.

In figure 2*b*, we display sample trajectories for these possibilities, demonstrating that in the case of the social interaction model, we are likely to observe clustered departures towards the foraging site. As shown in figure 2*b*, we observe qualitative and quantitative differences in the pattern of how the number of foragers changes as a function of time. Such patterns, together with movement trajectories of males, can be used to distinguish whether males on the lek forage independently of each other, or exhibit social interactions while foraging.

### (b) Directions for model extensions and linking with data

We now discuss some potential future directions for using such models and how to link such models with data (also see table 1).

#### (i) Male coordination for chasing an intruder

As already argued in §2(c), when an intruder male enters the lek, males on the lek may chase away this intruder on their own. Alternatively, more than one male on the lek may coordinate their movement to chase away the intruder. We can simulate these alternative scenarios by extending the agent-based model and simulations by introducing an intruder male. We can arrive at how model predictions of male movements differ between the aforementioned alternative chasing scenarios. We show two example sketches (not model runs) in figure 2*c*: if males react independently, we expect to find a substantial time difference in initiating the chase. However, if they are coordinating to chase away intruders, we expect that their trajectories would be highly correlated (or synchronized).

**Figure 3.**
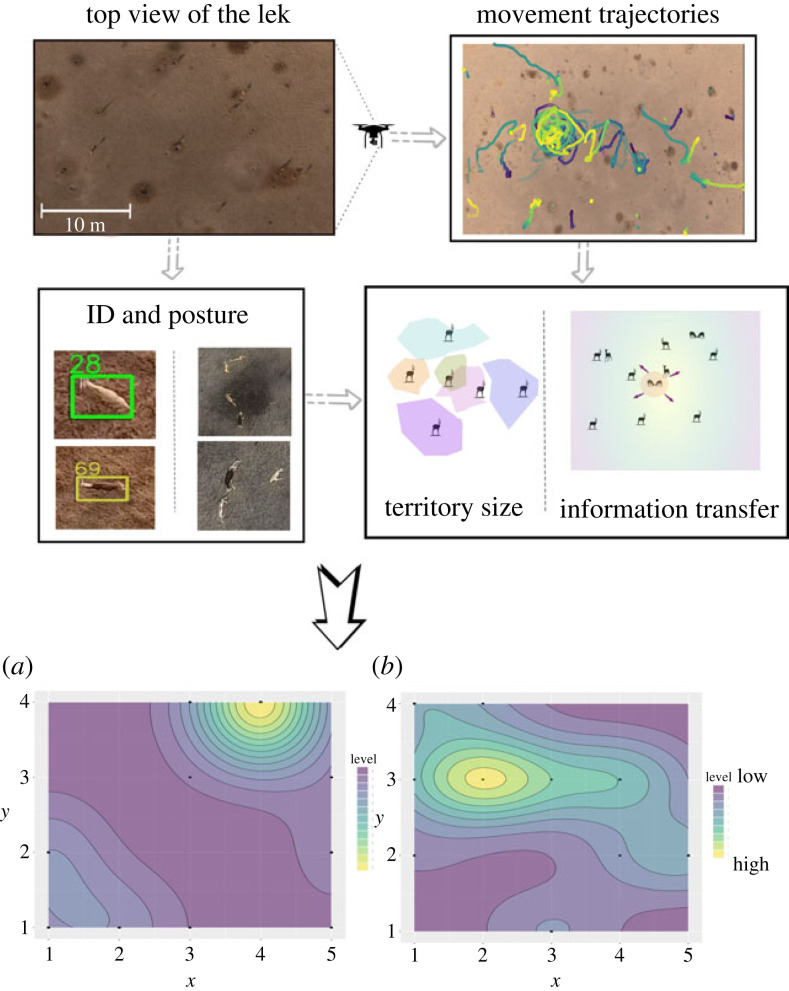
A preliminary analysis example from a sample blackbuck lek video clip. From the tracked video, we obtained a fine-scale spatio-temporal map of the interactions and activities of both males and females: (*a*) female presence heat map and (*b*) male aggression heat map. (Online version in colour.)

#### (ii) Female mate-sampling strategies

Next, we argue that agent-based simulations can be used to explore the relationship between female sampling strategies and their movement behaviour. As discussed in §2(c), female mate-sampling strategies influence, and are influenced by, movement patterns and local interactions. Conversely, we argue that the mechanisms of female choice (or mate-sampling strategies) can be inferred from the analysis of fine-scale movement patterns and local interactions. We illustrate this with the following four possible scenarios (see figure 2*d*):

1. Random mate sampling: Here, females do not have a preference for any male, and hence, this serves as a null model. Females move randomly between territories before mating.
2. Best of ‘*N*’: If a female’s strategy is to choose the best male out of the options available to her, we would observe that she samples many males before she mates. Therefore, we expect that a female returns to a territory for mating after sampling many territories.
3. Threshold approach: If a female mates with a male that is ‘good enough’, i.e. meets the threshold quality, she would mate with a male as soon as she finds a male that suits this criterion. Therefore, we do not expect revisits to any territory.
4. Conspecific cueing or choice copying: When a female moves on the lek, she might take visual cues from other females and copy the choice of other females to reduce sampling costs. In this case, we might see that a female’s movement trajectory or courtship choice is influenced by another female’s movement or courtship choice.

A possible hypothetical trajectory for each of the above four scenarios is sketched in figure 2*d*. To rigorously test these scenarios, we suggest extending the spatially explicit agent-based models shown above and making quantitative predictions on the properties of trajectories. One can then use high-resolution video recordings to test these predictions.

## 4. Illustration using blackbuck leks

We explain our proposed approach and the feasibility of data collection to implement this approach with an example study of blackbuck (*Antilope cervicapra*) leks [54–56]. Blackbucks are antelopes native to the Indian subcontinent. They mainly occupy grassland habitats. Females typically live in herds, while males exhibit a variety of grouping behaviours. While some males join herds, others may move solitarily or even form territories restricted to a small area. Blackbucks generally exhibit resource defence polygyny, where males occupy resource territories and females visit them for mating. However, in some populations, males exhibit lekking as an alternative mating strategy [15,55].

A high-resolution view from the top of the lek enables us to observe a reasonably large fraction of the entire lek area. From such aerial videos, we can, in principle, extract behavioural and spatial information such as movement trajectories of individuals, locations of territories, identity and postures of individuals and interactions between individuals. We demonstrate some basic analyses in the following sections.

### (a) Data collection

We recorded lekking behaviour using unmanned aerial vehicles (DJI Phantom-4 Pro) using an in-built camera on the drone, at a high spatial resolution (4k pixels covering an area of approximately 5000 m^2^) and at a fine-scale temporal scale (30 frames per second). The high spatial resolution allows us to digitally zoom in and detect the sex of each individual. The high temporal resolution allows us to track their movement trajectories and quantitatively analyze important behaviours such as intrusions by satellite males, mounting and tactile fights.

### (b) Data processing

We present preliminary analyses of trajectories and interactions of the blackbuck lekking system from a sample clip that is around 10 min in duration. We note that extracting information such as individual IDs, territory locations and movement trajectories from the high-resolution videos using the standard image processing tools is challenging because of the highly heterogeneous background. To overcome these difficulties, we adopt a visual detection and tracking open source package MOTHe, developed by our team [57]. This algorithm uses a convoluted neural network that is relatively easy to use and flexible enough to work even under heterogeneous backgrounds and lighting conditions.

First, we track individuals and mark territories using MOTHe [57]. This provides us with the spatial locations of all the territories. From these, we compute the distance between territory centres and the individual’s distance from the territory centre. We then manually monitor the video and note down the activity time budgets from this sample clip at 30 s intervals. At each sampling point, we observe the individuals for 5 s and note their behaviour, sex and territory location. For this example analysis, we used the following set of behaviours for the observations: (1) *R*: resting or stationary; (2) *W*: territory maintenance activities such as marking or walking; (3) *D*: courtship-related activities, mounting, display; (4) *C*: chasing away another individual; (5) *F*: tactile fights, and (6) *P*: parallel walks.

### (c) Data analysis

As argued earlier, the outcome of mating interactions for both males and females can be a function of local neighbourhood interactions. For example, females are thought to prefer areas on the lek that have fewer aggressive interactions, which are typically driven by male–male competition. This is also known as the harassment avoidance hypothesis [49,58]. In this context, we explore the relationship between the time spent by females on territories and male–male aggressions on these territories from our high-resolution blackbuck lek videos. We calculate the time spent by females on the territories as well as the frequency/degree of male aggression (fights and chasing behaviours) on these territories (see electronic supplementary material for more details). On the basis of figure 3, obtained from the analysis of a short sample clip, we find that females do appear to avoid areas of high male aggression, consistent with the harassment avoidance hypothesis. Of course, we clarify that this is only a preliminary illustration using a short clip. A statistically valid conclusion requires the analysis of many such sample videos. However, the methods demonstrated earlier—using high-resolution data collection and tracking approaches—potentially offer novel approaches to test different hypotheses or patterns, functions and evolution of lekking behaviour, e.g. as outlined in table 1.

## 5. Conclusion

In this article, we argued that spatial scales and dynamics implicitly play a key role in various hypotheses of lek evolution, and more broadly, on lekking dynamics. In addition, we demonstrated that a spatially explicit approach lays bare multiple factors that can contribute to lekking dynamics at different scales. We proposed that the principles and techniques from the field of collective behaviour can offer a unique perspective to our understanding of the proximate and ultimate dynamics of lekking. We showed some promise for this framework via an agent-based model and an illustration of blackbuck leks. Further, interactions on the lek typically change over time, in contexts that range from male–male competition, and male–male cooperation as well as matings, potentially altering many larger-scale patterns. Therefore, studying lekking systems at fine spatial and temporal resolutions may potentially help us better understand the strategies of female and male movement, and interactions within and between the sexes at various spatial and temporal scales, thereby offering new insights on both proximate and ultimate factors shaping lekking systems.

By using agent-based models, we illustrated how certain large-scale patterns emerge out of simple interactions. For example, attraction to a breeding ground and site fidelity together with local repulsion among males at the beginning of the breeding season could explain large-scale lek formation. To explain (coordinated) foraging episodes, we need to assume that local interactions change over time: e.g. individuals may switch to local copying of others’ behaviours together with foregoing site fidelity, albeit temporarily. We could also make testable predictions on how random versus synchronized departures differ. Broadly, we argue that studying fine-scale movement patterns and interactions opens doors to a holistic understanding of the mechanisms of lek formation, mate selection and intrasex competition among individuals on a lek. With these examples, we argue for future avenues of research that could utilize fine-scale movement information to explore the relative contributions of intrasexual competition and mate choice. For example, secondary mate-choice tactics—fidelity or preference for certain mating sites (central territories in many species) or copying the choice of others—can affect the opportunity for free mate choice for females. While the mate-choice copying mechanism is thought to be an important factor driving the mating success skew on leks, intrasexual aggression and competition among females are rarely explored.

Going further, we need to develop conceptual, theoretical and computational models to make spatially explicit predictions to disentangle various mechanisms that contribute towards lekking dynamics. Such methods in combination with high-resolution spatio-temporal data that cover movement and interactions on the whole lek will potentially shed light on the drivers of sexual selection in lekking systems. We hope this approach inspires a new direction of research in the field of lekking systems and more broadly in the area of sexual selection (box 2).

Box 2. **Key areas for future research.**

(a) *Models*: Develop spatially explicit models of lek formation and the mate selection process on leks. These could start with rules for local interactions and the movement of males and females. One may use simulations and/or develop analytical models to make predictions on the formation, patterns, maintenance and evolution of leks.
(b) *Technology for data collection*: To test models and hypotheses on fine-scale dynamics of the lekking system, we need high-resolution spatio-temporal data of lekking systems. In study species such as blackbuck, lekking grounds are spread over large areas. Therefore, to capture lekking dynamics over the entire lek, we may need multiple drones synchronously recording different parts of the lek. These recording sessions will be done over multiple days, under different conditions. Therefore, a variety of technical challenges will need to be addressed—starting from synchronous recording, handling large amounts of data, and detecting, identifying and tracking a large number of animals over multiple sessions, under different lighting and habitat conditions.
(c) *Testing spatial hypotheses for lekking dynamics*: With high-resolution spatio-temporal data, we can begin to look at lekking systems in a fundamentally different way. We can tease apart processes occurring at different spatial scales (e.g. hot-spot and black-hole hypothesis), and we can test assumptions of various models where spatial dynamics and interactions are important (do females visit territories on the lek randomly, etc., as demonstrated in the case study section?). Unlike the collective motion system that we took inspiration from, there is a multitude of interactions occurring at different spatial and temporal scales on a lek. Therefore, analysis of individual movement trajectories to infer interactions among individuals will likely require substantial modifications or the development of newer methods.

## Data Availability

Relevant codes and data are available via Github repository: https://github.com/tee-lab/lekking-collective-beh Two sample video files are available at Figshare: https://doi.org/10.6084/m9.figshare.20484366.v1. The data are provided in electronic supplementary material [59].
